# Study protocol for a cluster randomised controlled trial to assess the effectiveness of user-driven intervention to prevent aggressive events in psychiatric services

**DOI:** 10.1186/s12888-017-1266-6

**Published:** 2017-04-04

**Authors:** Maritta Välimäki, Min Yang, Sharon-Lise Normand, Kate R. Lorig, Minna Anttila, Tella Lantta, Virve Pekurinen, Clive E. Adams

**Affiliations:** 1 Department of Nursing Science, Faculty of Medicine, University of Turku , Turku, Finland; 2 School of Nursing, Hong Kong Polytechnic University, Hong Kong, China; 3 Turku University Hospital, Turku, Finland; 4 West China Research Center for Rural Health Development, Sichuan University Huaxi Medical Center, Sichuan University of China, Administration Building, No 17,Section 3,Ren Ming Nan Lu, Chengdu, Sichuan China; 5 Department of Health Care Policy, Harvard Medical School, 180 Longwood Avenue, Boston, MA 02115-5899 USA; 6 Department of Biostatistics, Harvard T.H. Chan School of Public Health, Harvard Medical School, 180 Longwood Avenue, Boston, MA 02115-5899 USA; 7 Department of Medicine - Med/Immunology & Rheumatology, Stanford University, 1000 WELCH RD. #204, Stanford, CA 94305-5755 USA; 8 Institute of Mental Health, Division of Psychiatry, University of Nottingham, Jubilee Campus, Wollaton Road, Nottingham, NG8 1BB UK

**Keywords:** User-driven intervention, Patient-centred, Psychiatry, Aggression, Treatment culture, Cluster randomised, Controlled trial, Effectiveness

## Abstract

**Background:**

People admitted to psychiatric hospitals with a diagnosis of schizophrenia may display behavioural problems. These may require management approaches such as use of coercive practices, which impact the well-being of staff members, visiting families and friends, peers, as well as patients themselves. Studies have proposed that not only patients’ conditions, but also treatment environment and ward culture may affect patients’ behaviour. Seclusion and restraint could possibly be prevented with staff education about user-centred, more humane approaches. Staff education could also increase collaboration between patients, family members and staff, which may further positively affect treatment culture and lower the need for using coercive treatment methods.

**Methods:**

This is a single-blind, two-arm cluster randomised controlled trial involving 28 psychiatric hospital wards across Finland. Units will be randomised to receive either a staff educational programme delivered by the team of researchers, or standard care. The primary outcome is the incidence of use of patient seclusion rooms, assessed from the local/national health registers. Secondary outcomes include use of other coercive methods (limb restraint, forced injection, and physical restraint), service use, treatment satisfaction, general functioning among patients, and team climate and employee turn-over (nursing staff).

**Discussion:**

The study, designed in close collaboration with staff members, patients and their relatives, will provide evidence for a co-operative and user-centred educational intervention aiming to decrease the prevalence of coercive methods and service use in the units, increase the functional status of patients and improve team climate in the units. We have identified no similar trials.

**Trial registration:**

ClinicalTrials.gov NCT02724748. Registered on 25^th^ of April 2016.

## Background

Mental disorders are among the most frequent and most disabling non-communicable disorders. Every year over 38% of the total European Union (EU) population suffer from mental disorders [1], and the lifetime prevalence rate is 25% [2]. Mental health conditions are the one of the most dominant contributors to the global economic burden of non-communicable diseases, and schizophrenia constitutes the second greatest global burden in terms of disability [3]. Schizophrenia is a severe mental disorder characterised by profound disruptions in thinking, affecting language, perception, the sense of self and impairing functioning through the loss of an acquired capability to earn a livelihood [4]. However, schizophrenia is a treatable disorder: there is strong evidence that antipsychotic medication and family psychoeducation can improve outcomes for people with a diagnosis of schizophrenia [5]. At the same time, challenging behaviour in people with serious mental disorders is an international concern. Although contradictory opinions exist in the literature, the association between an increased risk of violent behaviour and mental disorders has been documented [6]. In regards to lifetime violence, a significant association has been found with diagnosis of schizophrenia and other psychotic conditions [7]. It is also known that patients’ violent behaviour decreases physical [8] and psychological health among staff members in psychiatric inpatient care [9].

Discussion about the treatment of patients with aggressive or self-harming behaviour is frequently accompanied by an ethical conflict between patients’ autonomy on one hand and the requirement to prevent harm on the other [10]. The proportion of people who experience different coercive measures is relatively small and numbers are decreasing [11–13]. However, frequencies of compulsory admission [14] and other types of coercion vary across the EU [12]. Still seclusion, physical restraint and forced medication are used in many psychiatric hospitals [15, 16]. According to the recent health statistics in Finland, out of all people treated in in-patient psychiatric care in 2014 (*N* = 25,552), 14% (3,329 patients) were exposed to coercive methods [17]. Of those, 46% (1,520 patients) were isolated in seclusion rooms, 21% (696 patients) were tied to a bed with special belts in seclusion rooms (limb restrains), 24% (787 patients) received forced injections, and 10% (326 persons) were physically restrained [17]. With regards to the burden on families, relatives have experienced that involuntariness in patient care has been associated with the feeling of being excluded from treatment participation [18]. From the perspective of patients, their experiences of coercion are mainly negative [19–22]. As an outcome of coercive measures, it was found in Finland that the use of coercive measures was associated with increased mortality of acute psychiatric hospital patients [23].

Less coercive measures and restrictive techniques are being recommended for managing patient aggressive behaviour [24]. Some actions have already been taken to decrease seclusion and restraint [25–29]. Putkonen et al. [27] found that, in state hospital wards for men with schizophrenia and violent behaviour, seclusion and restraint could be prevented with staff education and without an increase of violence. Some evidence has also shown that less restrictive interventions, such as cognitive skills programmes [30] or short-term risk assessment [31], have some effects in decreasing the number of aggressive incidents on psychiatric wards.

Still, use of less restrictive interventions to manage aggressive patients is controversial [32]. Institutional organisation and clinical responsibility has traditionally aimed to provide a structured and safe environment for patients, to facilitate and monitor their treatment processes [33]. However, a clinical trial in Brazil by Huf et al. [34] found some limited evidence that using less restrictive measures did not harm patients, such as increase overall time of being restricted. Bergk and colleagues [35] in their clinical trial in Germany showed that patients did not show a clear difference between coercion experiences after seclusion or mechanical restraint use although contrary experiences have also displayed with low quality study methodology [36].

Despite the controversy of this topic, there is a need to establish, in collaboration with various parties, humane evidence-based interventions for prevention of aggressive behaviour among psychiatric patients [37]. Critical assessment of the content of patient treatment, as well as the consideration of patients’ and family members’ voiced opinions and concerns, are too often neglected by services, even though they are included in modern treatment agendas [38]. Procedures used for dealing with patients’ behavioural problems are still largely based on the needs of staff and are untested in comparative studies [24]. On the other hand, if new interventions are being used, descriptions of the content of the new interventions are often incomplete which hinders their implementation in practice [39–41]. There is also a lack of knowledge, which elements of interventions or programmes are effective or which mechanisms or processes have a real impact [37]. In addition, staff members may have their own untested traditions how to manage patients’ challenging behaviour on the wards because they are not always sufficiently aware which interventions are more cost-effective [42, 43] or are more effective, particularly in managing aggressive patient behaviour [40, 44] than others. Finally, the understanding of aggressive patient events is still unclear and there is a lack of studies investigating causal associations between risk factors and patient violence [45].

Positive experiences of making a difference in a wider context can also be found. The recent large-scale trials covering wide geographical areas in the United Kingdom [46–49], South Africa [50] and Switzerland [51] have encouraged us to design and conduct a nation-wide cluster randomised clinical trial to test the effects of user-centred and collaborative intervention to prevent and decrease coercive events in psychiatric hospitals. Contrary experiences of the impact of large-scale intervention trials also exist. Thornicroft et al. [52] found in their study (64 generic and specialist community mental health teams) that the Joint Crisis Plan (JCP) was not significantly more effective than usual treatment. One reason was that the JCP was not fully implemented in all study sites, and it was combined with routine clinical review meetings, which did not actively incorporate patients’ preferences. Other studies have also found problems in new working methods, which may hinder their use in clinical practice. These problems include, for example, that interventions have been difficult to adhere to, use and to adopt into routine care [53]. Other hindrances related to the assessment of the new methods have been slow patient recruitment [54] and sudden changes in local mental health policies [35]. In Norway, Aakhus et al. [55] examined in a cluster randomised trial with 80 municipalities, whether adherence to treatment guideline recommendations for elderly patients with depression could be approved by targeting healthcare professionals, patients and administrators. They found that the effectiveness of tailored intervention in implementing recommendations for elderly patients with depression in primary care was uncertain. Further, a one-year team-level intervention (14 teams in the REFOCUS group, 13 in the control group) did fail to coach staff behaviour toward a positive partnership between staff and patients with psychosis [56].

Although treatment systems in psychiatric hospitals have already developed toward having more positive approaches [57], the importance of developing interventions and making ward atmospheres more active and caring should not be forgotten [37, 58–60]. Recently Boumans et al. [37] described a reduction in the use of seclusion in a psychiatric setting. Patients and families worked together, in close collaboration, which included cyclic evaluation and readjustment of the treatment and a nurse care plan. Implicit, positive changes were found in the team process, such as increased interdisciplinary collaboration, team cohesion, and professionalization. Our previous small-scale project (funded by the Finnish Work Environment Fund, 111298) also identified the need and promises in improving ward atmospheres in psychiatric hospitals by educating staff members toward close collaboration between patients, relatives and staff members [20, 61–64]. In the randomised controlled trial (RCT), we will also use user-driven approach [65] that includes, close collaboration with patients, staff, and family members in implementing the intervention in clinical practice and listening and accounting for their preferences and needs in daily practice [66]. The focus of the approach will therefore be bottom-up rather than top-down [67]. However, this requires stronger effort in our intervention in organising meetings, discussions and workshops with end-users to ensure everyone is aware of end-user needs and preferences.

In this study, we will implement an educational intervention for nursing staff to improve treatment culture on psychiatric wards and support team climate in staff members in psychiatric hospitals with a nation-wide cluster trial to test the effects of the educational intervention to decrease coercive incidents in psychiatric hospitals.

## Methods

### Aim of the study

To compare the effects of an educational intervention to usual practice (standard care, no specified staff education) on improving treatment culture and supporting team climate in staff members, which further could reduce the need for the use of coercive methods in psychiatric care. Although the intervention is designed to impact the daily practice and treatment culture of each hospital organisations, our attempt is also to improve outcomes at the level of the individual patient and nursing staff. Therefore, the outcomes of the study will be assessed from the organisational, patients’, and staff members’ points of view.

The primary objective is to investigate whether the educational intervention for staff will decrease the incidence of patient restrictions - specifically use of seclusion rooms on psychiatric wards of hospitals. The secondary objectives are to investigate whether the educational intervention changes the incidence of limb restraint, forced injection, physical restraint and service use in psychiatric hospitals. The study will also investigate whether the educational intervention for staff members changes patients’ functional capacity, level of treatment satisfaction and quality of life. From the point of view of the nursing staff, the study will investigate whether the educational intervention effects team climate and staff turn-over.

### Trial design

Our study is a single-blind, two-arm, stratified cluster randomised trial.

### Setting and sample

This is a nation-wide study to be conducted across Finland in hospitals with psychiatric beds. Finnish mental health services are arranged by municipalities (*N* = 317 [68]), each individual municipality forms joint municipal authorities with other municipalities, or hospital districts, and are supplemented by private and third sector services (e.g. associations and foundations). People in need of inpatient psychiatric care are treated in general health services (e.g. health centres) or specialised medical care services organised by hospital districts [69]. This study will be conducted in hospitals with psychiatric beds in the non-private sector. Hospitals as cluster will be recruited and randomised.

Inclusion criteria of required participating hospital organisations are: Finnish-speaking, to have at least 1 psychiatric ward, open 24 h a day, seven days a week, and, when necessary, are able to use coercive measures defined in the Finnish Mental Health Act (1116/1990, [70]) (seclusion room, limb restraint, forced medication, physical restraint). Wards will be excluded if they are specialised in forensic, psychogeriatric, or child and adolescent mental health care alone or if a similar type of project is underway or is planned to start there. In total there are 28 eligible hospitals. Once a hospital is allocated to any comparison arm, all health care professionals working in wards of the hospital and patients admitted to those wards are eligible to participate to the study. Based on the Finnish health statistics of the National Institute for Health and Welfare [17], patients with a primary diagnosis of schizophrenia represents the second most common diagnostic group in psychiatric hospitals, after people with depression or recurrent depressive disorder [17]. People with schizophrenia are treated in psychiatric hospitals if they are in an acute phase and need for specialized care: 60% of all hospitalised patients in 2014 were hospitalised through an emergency admission [17].

## Study procedure

### Recruitment and randomisation

Written information letter describing the study are to be sent to the administrators of each hospital organisation. They are given up to four week-time to show their preliminary interest and willingness to participate. This allows an adequate time for them to think through their decision and ask any questions related to the study.

Hospital organisations, within which there will be eligible wards, agreeing to participate will be randomly allocated to either receive staff education intervention (intervention group) or not receive staff education (passive control group). This allocation is organised by the research group as soon as baseline information of the wards is collected. The unit of randomisation is the hospital organisation within which there will be psychiatric wards. The cluster design will be used to avoid contamination in intervention effects between individual staff members. We will use centralised randomisation at the University of Turku (Department of Mathematics and Statistics) stratified by number of patient beds and nursing staff to assure as similar number of participants as possible in the intervention group as in the passive control group. Randomisation will be fully concealed and computer-generated by an independent statistician, who is not involved in the study. Investigators enrolling wards will not be able to foresee the assignment.

Data analysts will be kept blinded to allocation. Due to the type of intervention, allocation will be unmasked after randomisation to patients and their relatives, contact persons on each ward, health care staff delivering patient care on the wards, and the researchers participating in the intervention design and implementation; this will reflect real-world care. The Data Management Committee (DMC) will undertake ongoing safety surveillance. Investigators running the preliminary analysis for the DMC will be masked to data until investigators release the database. In addition, the statisticians and the National Register holder [71] responsible for Finnish routine data used in this study will be masked to ward allocation and patient data in each arm.

Local medical registers in each study hospital will form the data base of the study. For patient survey, staff in sampled hospitals will recruit every Finnish speaking patients and over 18 years admitted and treated on the wards during the patient data collection period before their discharge to achieve a sufficient sample of patients in anonymous survey. Inclusion criteria are patients aged over 18 years, of either sex, on discharge from a psychiatric hospital, are able to use the Finnish language, and are able to participate in the study based on their free will. Returned completed questionnaire will be interpreted as a voluntary participation in this study. We will include no formal test of capacity but will rely on the judgment of experienced health care professionals in their routine assessment, when nearing the point of discharge. Further formalized assessment is not part of routine care.

Patients who are under 18 years, incapable to use the Finnish language or judged to be not capable to participate in the study based on their mental status will be excluded.

**Fig. 1:**
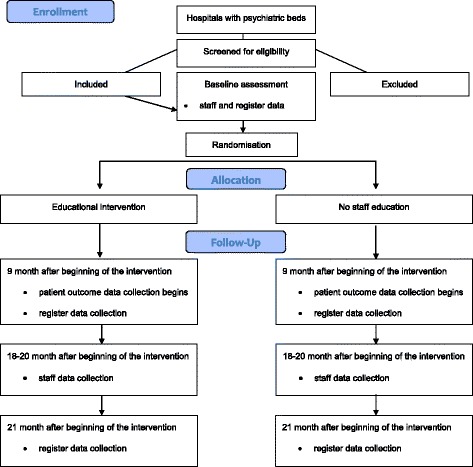
Protocol flow diagram

The flow of the study is described in Fig. 1. Flow diagram.

## Study interventions

### Intervention units

The overall aim of the intervention is to encourage collaborative practices between staff, patients and family members that will lead to the adoption of a less coercive treatment approaches in the ward. The intervention is designed to impact treatment culture and thereby treatment practices on the study wards. We hope to improve outcomes at the level of the organisation, patients, and staff members. To achieve the study goals we will support skill developments, intellectual resources, motivation and encouragement of staff members to make practical changes on the ward practices.

A pilot study has been undertaken with staff members, patients and relatives in one hospital ward not included in the study. This ensured acceptability of the intervention, readability and usefulness of all steps and of the educational content and materials, acceptability for the target population, understanding of the messages, coherence of the programme and feasibility of the time schedule. Based on the pilot, the intervention includes components based on Glasziou’s and Haynes’ [72] pathway to improve health outcomes:

1. Staff education: identification of problems in current treatment practices, and analysis of the local house rules and quality of the service facilities (involves interviews with patients, families and staff) [73]. The quality gaps and areas to be developed will be described and future steps for the development will be decided. Knowledge about evidence-based studies how to fill possible quality gaps will be shared with staff members, and strengths, weaknesses, opportunities, and threats (SWOT) related to the future changes will be captured. (**Acceptance**)
2. Local meetings: involvement of staff members, patients, relatives, and the trial team to specify detailed areas to be developed and the specific steps to be taken. Possible barriers and facilitating factors for change will be identified. (**Applicability**)
3. Shared information packages: intervention materials to support staff’s competence will be made available. (**Available**)
4. Support available from the project team: monthly monitoring and support calls or emails as to prompt and encourage change in staff members. (**Able**)
5. Implementation: hands-on-support provided by the trial team. The contact persons will work with the staff to help them gain confidence in the new ideas of the intervention. The understanding of the intervention will be reviewed (Interim Evaluation). (**Acted on**)
6. Identification of the possible change: the use of coercive methods and house rules will be analysed, and possible differences in situation in baseline and after intervention, such as practices, and treatment methods and associated with patient care will be analysed and shared with staff members. (**Agreed on**)
7. Awareness of engagement and motivation: treatment practices and outcomes will be monitored and evaluated by the trial team. The team, working with the contact persons on the wards, will, in collaboration with staff members, assess how specific intervention fidelity criteria have been fulfilled [74]. (**Adhered to**)

A summary of the intervention is described in Table 1.

**Table 1:** Description of the intervention according to the TiDieR (modified based on Hoffman [99])

Categories	Description of the intervention
Name	Educational intervention to support positive treatment culture and team climate in staff members in psychiatric hospitals
Rationale/Theory	Patients’ conditions, treatment environment and ward culture may affect patients’ behaviour. Use of coercive methods could be prevented with staff education about user-centred if more humane approaches as well as collaboration between patients, family members and staff members could be increased. Staff education may further positively affect treatment culture and lower the need for using coercive methods in psychiatric hospital care. [46–51]
Materials	Information about evidence-based research, written information package of intervention materials, and monitoring tools.
Procedures	Identification and analysis of current treatment practices, local house rules and quality of the service facilities. Identification of quality gaps, SWOT, barriers and facilitators for change. Dissemination of research evidence. One-day workshop seminars, local meetings and outreach visits. Ongoing monitoring and support by calls/emails.
Providers	Trial team: with a background of psychiatric care as nurses/researchers, an academic qualification (master and/or doctoral level) with an experience in continuing education of staff members (professor, senior researcher, project researcher, doctoral student, master students). Staff members: different health care professionals.
How	Face-to-face seminars with lectures, workshops, group meetings, outreach visits, telephone and email contacts with staff members. If needed, video meetings will be organized with staff members.
Where	At the psychiatric wards and at the University facilities (workshops, seminars).
When and how much	Intervention will take 18 months: - Identification, analysis and sharing current treatment practices, use of coercive methods, local house rules, and quality of the service facilities (months 1–4) - Identification of quality gaps, SWOT analysis in workshop and local meeting, barriers and facilitators for change in each ward, and dissemination of research evidence in workshop; one-day workshop seminars, local meetings and outreach visits (months 5-8), - Ongoing support by calls/emails provided, workshop, local meetings (months 9–18).
Tailoring and modifications	The education process with specific protocol is similar at each ward. The activities taken on each ward based on the need analysis and the quality gaps may be tailored to fulfil the needs of each ward.

### Comparison unit

Wards allocated to the control arm (standard care, passive control group) will continue with their usual care, and staff will not receive additional education offered by the trial team. There are no restrictions on how nursing staff work in these wards, although participation in corresponding projects is not supported. Any workshop seminars, local meetings, outreach visits or structured monitoring or support will not be organised in the comparison wards. As in the intervention hospital organisations, any analysis of the current treatment problems or possible quality gaps to be shared with the staff members will not be collected or shared by interviews or observations. Any information packages aiming to support their positive treatment culture will not be shared with the staff members working in these hospital organisations. The trial team will contact the control wards only to collect baseline data of the wards and the outcome data. After the 18 months intervention and follow-up period, comparison wards will be given a possibility to participate in workshops and seminar related to the intervention.

## Measures

### Background information

Patients: The demographic information of the patients (age, gender, marital status, educational level, housing, employment status, number of psychiatric hospital treatment periods, the time of first contact with psychiatric services) will be collected as part of a patient follow-up survey.

Demographics of unit of randomisation: The following information about the units will be described: a number of hospital beds, a number of patients, bed/patient ratio, a number of treatment periods, an average length of stay, gender ratio, patient average age, three most common diagnose (ICD-10) [75], and a number of staff/professional group. In addition, a short description of the ward type and treatment methods will be asked.

## Primary outcome

### Organisational outcomes

The primary outcome of the study is the incidence of seclusion room use by patients in each unit. This will be assessed based on local (and national, if possible) health registers (Finnish Mental Health Act 1116/1990, [70]). The outcome will be measured at three time points: baseline (in 2015) based on local register (at the end of 2016), year 2016 (at the beginning of 2017), and year 2017 (at the beginning of year 2018). Information collected from each organisation is comparable across hospital organisations. The national register holder, Care Register for Health Care [71] gives instructions on how the information regarding treatment notifications shall be collected, recorded and sent annually to the National Institute of Health and Welfare. To ensure that the information about incidence of seclusions can be obtained in full detail, data will be collected either manually based on nurses’ notes and/or medical registers at the local organisations.

## Secondary outcomes

### Organisational outcomes

*Other types of coercive measures*: incidence of other types of coercive measures used on patients (limb restraint, forced injection, physical restraint) according to the Finnish Mental Health Act (1116/1990, [70]) (yes/no), including the frequency and/or length (in minutes, if possible) of these measures. The outcome will be measured at three time points: baseline based on local register from year 2015 (at the end of 2016), year 2016 (at the beginning of 2017), and year 2017 (at the beginning of year 2018). Information to be collected in each organisation will be comparable based on the instructions of data recording by the National Institute of Health and Welfare. The data will be collected either manually based on nurses’ notes and/or medical registers at the local organisations.

*Service use* (collected from local and/or national registers): type of admission, length of stay (days), death [yes]). The data will be collected either manually based on nurses’ notes and/or medical registers at the local organisations. Information to be collected in each organisation is comparable based on the instructions of data recording by the National Institute of Health and Welfare [71]. The outcome will be measured at three time points: baseline based on local register from year 2015 (at the end of 2016), year 2016 (at the beginning of 2017), and year 2017 (at the beginning of year 2018).

### Staff outcomes

*Team climate* among nursing teams (Team Climate Inventory, TCI, 38 items [76, 77]) will be surveyed at baseline and 18-20 months after beginning of the intervention. The inventory consists of four subscales; participative safety, support for innovation, vision and task orientation. Items in subscales of participative safety and support for innovation are measured with 5-point Likert-type scale, while subscales of vision and task orientation are measured with 7-point Likert-type scale. Higher scores indicate better team climate [77]. In the Finnish population, subscales of the inventory have demonstrated high internal consistency (Participative safety α = 0.88; Support for innovation α = 0.87; Vision α = 0.95, Task orientation α = 0.91 [78]) and moderate correlations (r = 0.36-0.48) with other methods assessing team innovativeness [79].

*Turnover* will be measured at baseline (based on local register data from 2015), year 2016 (at the beginning of 2017), and year 2017 (at the beginning of year 2018).

### Patient outcomes

*The functional capacity of patients* (Global Assessment Scale, GAS [80]), rated by staff members at the time of the discharge process, will be assessed. The value of the measure can vary between 0 and 100. Higher scores indicate better functional capacity of patients [80]. The scale has demonstrated high inter-rater reliability (r = 0.72) and significant correlation (r = - 0.64) with other scales used to assess the symptom complexity of patients [81]. The measuring of this outcome will begin 9 months after beginning of the intervention (at the beginning of 2017).

*Patient treatment satisfaction* (Client Satisfaction Questionnaire, CSQ-8, 8 items [82]) will be assessed with a structured paper survey for every patient at the time of their discharge process. Patients respond to the questions using a 4-point Likert scale. Responses are scored from 1 to 4, and thus the possible total scores range from 8 to 32. Higher scores indicate greater satisfaction with treatment [82]. The survey has demonstrated a high level of internal consistency (Cronbachs’ α 0.92) and significant correlation (r = 0.67) to other methods used to measure treatment satisfaction [83]. The measuring of this outcome will begin 9 months after beginning of the intervention (at the beginning of 2017).

*Quality of Life* (Quality of Life Enjoyment and Satisfaction Questionnaire-Short Form, Q-LES-Q-SF, 16 items [84]) will be assessed with a structured paper survey for every patient at the time of their discharge process. Each of the 16 items is rated on a 5-point scale that indicates the degree of enjoyment or satisfaction experienced during the past week. Higher scores on indicate greater contentment or satisfaction [84]. In the Finnish psychiatric patient population, this instrument has demonstrated high internal consistency (Cronbachs’ α = 0.89) and moderate correlation with other methods used to assess quality of life (r = 0.445) [85]. The measuring of this outcome will begin 9 months after beginning of the intervention (at the beginning of 2017).

## Feasibility of the intervention

Feasibility of the intervention will be analysed from organisation, staff members, and patients’ point of views. Acceptability (willingness of patients to participate, satisfaction with the intervention, willingness to continue the intervention), implementation (degree of execution), integration (drop out), and practicality (easy to use, yes/no) [86], in daily practice will be assessed at the end of the study.

## Fidelity and quality components of the intervention

The fidelity of the intervention and staff adherence to the intervention will be supported and assessed (Table 2). Each item completed achieves a score of 0-1.00, giving a total percentage of 0%-100%. Lastly, the final seminar will be organised by the team in conjunction with the intervention units.

**Table 2:** Implementation stages and fidelity criteria of the process based on Glasziou and Haynes et al. [72])^a^

Implementation stage	Fidelity criteria
Acceptance A one-day workshop (*X*2) for ward managers and contact persons will be organised by the trial team; the results of the information collected at baseline will be shared; preliminary action plans for units will be designed; queries from the staff will be answered. A local ward meeting will be organised by the senior ward manager and a contact person on each intervention ward. House rules for patients will be collected and analysed from each ward.	At least one ward manager/contact person in each intervention unit will attend the one-day workshops (1^st^ and 2^nd^ workshop) (80%). The first local meetings will be organised and documented on each ward (100%). House rules will be analysed (100%).
Applicable A series of local meetings with staff members, patients, and relatives will be organised by the trial team; areas to be developed and specific steps to be taken will be identified; barriers and facilitating factors for change will be described; strengths, weaknesses, opportunities, and threats related to the educational intervention will be identified through a SWOT analysis.	The first outreach visit on each ward will be organised (100%). At least 50% of staff on each ward will attend the first outreach visit.
Available An information package of an intervention to support staff’s competence will be available.	An Action Plan for each ward will be developed (100%); the content of the information package will be shared with the staff (100%).
Able Monthly monitoring/support calls/emails by the trial team will prompt and encourage changes on the wards.	Ward managers/contact persons or senior ward managers will report the progress of the changes (including harms) by email/telephone (12 calls or email/12 months; 100%).
Acted on The trial team will visit each ward to give hands-on support to staff members, ward managers and contact persons so that they will gain confidence in implementing the new ideas on the wards. The Action Plan will be revised if needed. A one-day workshop for an Interim Evaluation Seminar will be organised.	The second outreach visit on each ward will be organised. At least 50% of the staff on the ward will attend the visits. The third workshop will be organised to review the implementation process; at least one person from each unit will attend (Interim Evaluation) (95%).
Agreed on Outcome assessment and house rules will be analysed by staff members and the trial team; possible differences in previous and current actions will be identified.	Patient coercive methods and house rules will be analysed (100%).
Adhered to Daily practices will be monitored by the trial team. The final workshop will be organised.	Daily practices will be monitored and outcomes of the intervention will be evaluated in a meeting on each ward. The third outreach visit on each ward will be organised. At least 50% of the staff on the ward will attend the visits. At least one senior ward manager/contact person in each intervention unit will attend (95%) the fourth workshop.

^a^Killaspy et al. [74]

The strengths, weaknesses, opportunities, and threats related to the new practices will also be discussed and categorised based on a SWOT analysis. Possible barriers and facilitating factors for change in each unit will be identified during workshops and outreach visits. Further, to gain a deeper understanding of staff members’ willingness to engage themselves in the new approach in practice, interviews will be conducted at the last phase of the study for staff members, patients and relatives. Their perceptions of human treatment environment with the following topics will also be assessed: a) Uniqueness of the individual; b) Real choices; c) Attitudes and rights; d) Dignity and respect; e) Partnership and communication; and f) Evaluating recovery-oriented mental health practice [73]. Convenience sample method will be used to recruit participants into the interviews (*N* = 50% staff members and patients from a unit and 25% patients’ relatives will be interviewed [73]). In addition, their opinions about the possible changes at the units will be discussed.

## Data management

All original paper and electronic data will be managed and held in accordance with the University of Turku and the national standards of the Finnish Personal Data Act (523/1999, [87]), partner organisations’ special requirements, and the health care organisations’ policies and national acts. The Council for International Organizations of Medical Sciences (CIOMS [88]) and the Declaration of Helsinki [89] will be followed, to ensure confidentiality of data. The material for the study will be mainly obtained from local registers, case notes and national health registers, and through patient and staff surveys. Data will be in paper and electronic format and saved electronically for statistical analysis. The qualitative data will be digitally recorded and transcribed to be analysed in written format. Members of the DMC are representatives from the ethical board, mental health associations, hospitals and hospital districts, national health and welfare institute, and statistics. Their role is to support, advice and evaluate the trial as well as intervention and outcomes. They also provide guidance related to financing, modifications, timing, risk management and quality assurance. The DMC have meetings twice a year.

## Power and sample size

We have systematically searched for, but found no directly relevant past work [31, 90]. We assume that if the data will be obtained from local hospital registers by sampling 7 clusters (hospitals) with 265 subjects each in intervention group and 7 clusters with 265 subjects each in control group, we will have 80% power to detect a difference between the group proportions of−0.0400. The control group proportion is 0.1100. The intervention group proportion is assumed to be 0.1100 under the null hypothesis and 0.0700 under the alternative hypothesis. The test statistic used is the two-sided Z test (unpooled) with an overall significance level at 0.05. We may assume based on the hospital registers that sample size for the total population admitted in the study wards in one year will be 3710. However, if we consider a loss of 20% patients in the local care registers, [91] the total number of patients on the randomised wards should be about 4454 patients. (PASS 11 software [92]). Further, if we assume 50% response rate for patient survey [93] out of possible 3710 participants, we will assume that we will obtain 928 filled questionnaires during 6 month survey data collection period. The sample size calculation was adjusted for intra-cluster correlation at 0.005.

## Data analysis

### Statistical analysis

The Data Management Committee including a statistician outside of the research team will verify the statistical analysis and the Quality Assurance Process.

The baseline characteristics of the units, and respondents (patients, staff members) will be summarised using descriptive analysis (mean [standard deviation], median [interquartile ranges] or proportions) and compared between each intervention group, respectively.

The analysis will be carried out on an intent-to-treat (ITT) basis. Assumptions of normality of the residuals will be investigated. For the primary outcome, incidence of coercion methods used at the baseline (year 2015), year 2016 (at the beginning of 2017), and year 2017 (at the beginning of year 2018) will be calculated. Possible differences in patient characteristics between the intervention and the control group at baseline will be tested by Chi-Square or t-tests based on type of variables. This analysis will help to decide whether baseline characteristics of patients should be adjusted for in the model for the primary outcome comparison after intervention. The hierarchical (participant nested within ward) Poisson model will be used to estimate and test the relative risk of being coerced in the intervention and the control group, which takes into account the length of staying of patient’s in hospitals. With the same principle, for secondary outcomes, a hierarchical linear model will be used for normally distributed variables such as patient satisfaction and team climate, a hierarchical logistic model for dichotomous outcomes such as any admission or any adverse event, and the Poisson model for hospitalisation stay lengths or time spent in a seclusion room. A sensitivity analysis will be conducted by comparing the primary data at the follow-up and the fidelity information [47]. If poor fidelity was found for the primary data, we may consider evaluating change rate of coercive methods use between the two groups using all three measures over time by applying repeatedly measured models with random effects of hospital clusters. The results from the secondary analyses will be treated as exploratory. Estimates and confidence intervals will be reported only. The CONSORT guidelines for randomised trials [94] will be followed throughout our data analysis and the reporting of our study results. This study protocol follows the recommendations of the SPIRIT Statement [95].

### Qualitative analysis

Qualitative data (SWOT analysis, possible barriers and facilitating factors for change, engagement in the new approach) will be analysed by thematic categorisation. A combination of deductive and inductive approaches will be used to gain an understanding of data. First, the taped interview data will be transcribed by researchers, which will be overseen by MV and MA to increase consistency, supervision and support the appraising process of the research group. The researchers will introduce themselves in the transcripts. Its content will be coded and further categorised by the specific themes by methods of Braun and Clarke’s [96] phases of analysis process. Further, the validity and reliability of the coding will be checked by MA and TL by recoding and defining categories from a random sample of pages of the transcripts. For the qualitative data, the COREQ checklist by Tong et al. [97] will be used for reporting.

## Dissemination

The Department of Nursing Science at the University of Turku in Finland is nationally and internationally known for its research related to aggression in mental health care. It is also one of the leading research centres focusing on preferences of patients and nursing staff within the field in Finland. The Academy of Finland, as a main funder, together with Turku University Hospital, the University of Turku, in close collaboration with Hong Kong Polytechnic University of China (SAR), is providing the research infrastructure for the study as well as opportunities to spread knowledge from this project locally and internationally in collaboration with the WHO Western Pacific Region. The results of the study will be published in international referee-based, high-impact scientific journals, national vocational journals, newspapers, seminars, conferences and on the project’s website and via social media campaign.

## Discussion

This is a large-scale randomised controlled trial investigating whether staff educational intervention is associated with a decrease, no change or even increase in use of coercive methods and patient services, a changed global state, treatment satisfaction and quality of life, and team climate among staff members. This is a single-blind, two-arm stratified cluster trial, involving hospitals with psychiatric hospital beds across Finland. Based on good examples abroad and extending educational interventions supporting relatives’ engagement in patients care, we believe that the study results may be beneficial in national and international contexts. Based on previous literature and our own experiences, we are prepared to face challenges in implementing and measuring the effects of the new intervention in mental health services and patient well-being [47, 53].

## Study status

To date, we have recruited 15 hospitals, in which a total of 28 psychiatric wards are willing to participate. Wards were randomised in May 2016 (13 intervention wards, 15 in comparison wards).

## Abbreviations

CSQ-8: Client Satisfaction Questionnaire
DMC: Data Management Committee
EU: European Union
GAS: Global Assessment Scale
ITT: Intent-to-treat
PASS: Power Analysis and Sample Size
Q-LES: Q-SF - Quality of Life Enjoyment and Satisfaction Questionnaire-Short Form
SWOT: Strengths, Weaknesses, Opportunities, Threats
TCI: Team Climate Inventory
WHO: World Health Organization
